# Food preferences and periodontal status of adults assisted by a public health care system

**DOI:** 10.1371/journal.pone.0291878

**Published:** 2023-10-18

**Authors:** Juliana Cristina dos Reis Canaan, Marcelo Martins Canaan, Patrícia Daniela Costa, Michel de Angelis Pereira, Paula Midori Castelo, Vanessa Pardi, Ramiro Mendonça Murata, Luciano José Pereira

**Affiliations:** 1 Department of Medicine, Universidade Federal de Lavras (UFLA), Lavras, Minas Gerais, Brazil; 2 Department of Nutrition, Universidade Federal de Lavras (UFLA), Lavras, Minas Gerais, Brazil; 3 Department of Pharmaceutical Sciences, Universidade Federal de São Paulo (UNIFESP), Diadema, São Paulo, Brazil; 4 Department of Foundational Sciences, School of Dental Medicine, East Carolina University (ECU), Greenville, NC, United States of America; National Dental Research Institute Singapore / Duke NUS Medical School Singapore, SINGAPORE

## Abstract

This study aimed to investigate the relationship between food choices and periodontal health status (PHS) in adults who receive care through a public health system. We evaluated food preferences and periodontal status in a sample of 442 individuals with at least eight natural teeth. We employed the Food Frequency Questionnaire (FFQ) to assess food choices and the Periodontal Screening and Recording (PSR) instrument to evaluate periodontal health status during clinical appointments. Fisher’s discriminant analysis was used to differentiate the participants according to PHS severity within three age-ranges (18–39; 40–59 and > 60 years-old). The results showed that the prevalence of overweight/obesity was high in all age groups (above 65%), and BMI increased with age, accompanied by an increase in the prevalence of chronic diseases. A lower preference for natural foods and a higher intake of processed and ultra-processed foods, along with a high waist circumference and diabetes, were associated with a poorer periodontal health status. In the 18–39 age group, a lower waist circumference was associated with healthier periodontal status. In the 40–59 age group, a worse periodontal status resulted from a higher frequency of diabetes, lower intake of green leafy vegetables, olive oil, and fruit, and higher intake of industrialized juice. Conversely, a healthier periodontal status was associated with a lower frequency of diabetes and higher intake of fruit and vegetables. In the > 60age group, the worst periodontal status was associated with male sex. Overall, the study highlights the possible beneficial role of a healthy diet in maintaining periodontal health, particularly for those who receive care through a public health system.

## Introduction

Oral diseases constitute an important public health problem worldwide due to the impact on quality of life and the high associated costs, although most of them being largely preventable [1]. In this context, periodontal disease stands out, as an often silent inflammatory condition that affects the supporting structures of the teeth such as the gum, periodontal ligament and bone [2,3]. It represents the interface of a systemic proinflammatory state, against the development of a pathogenic microbial biofilm on the gingival margin, intertwining it with metabolic and cardiovascular diseases [4,5]. Risk factors such as genetic predisposition [6], nutritional factors [7], smoking [8], suboptimal controlled metabolic diseases (diabetes and obesity) [9,10], poor oral hygiene [11] and certain psychosocial variables [1,12] are closely associated with the occurrence of periodontal disease, some of which are subject to modification.

Brazil is a country of continental dimensions, with a medium-high economy. However it presents relevant social and regional disparities [13,14]. Since 1988, the country introduced a public and comprehensive Unified Health System (UHS, denominated SUS in Portuguese), with emphasis on primary care on the community level, whose main pillar is the Family Health Strategy (FHS). It is executed by means of a multidisciplinary health team assisting a circumscribed population employing prevention approaches [15], professional appointments for spontaneous demands [16] and longitudinal follow-up and treatment of chronic diseases [17] including periodontal disease [18].

As a developing country, Brazil is experiencing a demographic and nutritional transition, with a substantial increase in the prevalence of non-communicable chronic diseases (NCDs), with a significant impact on morbimortality [19–21]. NCDs are closely associated with lifestyle which can often be modified by the adoption of healthier eating habits [20,22]. Diet, as well as deleterious behaviors related to excessive sugary drinks and alcohol consumption, have been reported as capable of predisposing the progression of periodontal disease and tooth loss [23]. Besides, micronutrient deficiencies, especially for vitamins C, D and B12, may contribute to periodontal disease onset and progression [24]. On the other hand, adherence to the Mediterranean or a semi-vegetarian high-fat diet seems to provide benefits to the periodontal health [25,26].

Determining the impact of diet in the prevention of NCDs such as periodontal disease, may subsidize preventive measures at the public health level. The possibility of introducing simple changes in food choices, especially considering low-income populations that do not have resources for healthcare is of paramount importance. Previous study from our group showed that higher consumption of omega-3, fiber, zinc, calcium, retinol, and riboflavin were associated to a better periodontal status in adults assisted by the UHS [27]. However, defining which foods in the diet are most associated with periodontal status can facilitate patients’ understanding and adherence to treatment. Besides, an improved diet plan may have positive adjunct effects on conventional non-surgical periodontal therapy [28] in the primary care program. Thus, the aim of the present study was to investigate the relationship among food preferences and clinical, behavioral, sociodemographic, and periodontal health status in adults assisted by a public health system.

## Material and methods

The study was conducted in the area covered by the Family Health Strategy (FHS) in the municipality of Lavras, whose population is estimated at 110 thousand inhabitants and located at latitude 21°14’43 south and longitude 44°59’59 west in the state of Minas Gerais, Brazil. According to the last survey of 2010, the municipality of Lavras occupies the 5th place in the ranking of the Human Development Index (HDI) of the state. The research was previously approved by the Human Research Ethics Committee of the Federal University of Lavras (COEP/UFLA, Minas Gerais, Brazil–under protocol number 85767618.1.0000.5148). We followed the “Strengthening the Reporting of Observational Studies in Epidemiology (STROBE)” guidelines for reporting observational studies.

Sample calculation and characteristics are detailed in a previous study [27]. In brief, the estimated prevalence of periodontitis was utilized, along with an absolute estimate precision of 5% and a significance level of 5% [29,30], leading to a sample size of 361 individuals. To account for potential losses and the variability in the number of individuals within each coverage region, this calculation was adjusted by 20%, yielding a minimum required sample size of 434 individuals. Participants were randomly selected among users of primary health care (52,628 individuals) from 17 Units of the FHS in the municipality. The inclusion criteria consisted of individuals of both sexes over 18 years of age, who had at least 8 natural teeth and who voluntarily consented to participate in the project. People undergoing recent changes in their dietary pattern were excluded.

Data collection was carried out through cross-sectional home visits. On the day of the visit, a Food Frequency Questionnaire (FFQ), clinical periodontal examination, anthropometric measurements and evaluation of clinical, behavioral and sociodemographic characteristics were applied. Information about food consumption was collected with the help of a photo album and the FFQ was adapted to the size of the portions [31,32]. The Food Frequency Questionnaire (FFQ), is a widely used dietary assessment tool that is used to estimate an individual’s dietary intake over a defined period of time, typically over the past month. The FFQ was applied as a self-reported questionnaire asking individuals to recall how often they consumed specific foods and beverages over the last month. The FFQ consisted of a list of commonly consumed foods and beverages, along with response options that indicate the frequency and portion size of each item consumed. The FFQ was applied to comprehensively capture the dietary habits of individuals. The data collected from the FFQ was used to estimate food choices patterns. The clinical periodontal examination of the volunteers was performed by only one trained and properly calibrated examiner, in a room with natural and appropriate lighting, using the Periodontal Screening and Recording (PSR) approach [33]. The mouth was divided into sextants and all teeth were evaluated (except third molars). The sextants were classified using an ordinal scale starting from healthy (score 0), bleeding on probing (score 1), presence of calculus (score 2), probing depth of 4–5 mm (score 3), and probing depth ≥6 mm (score 4). The Periodontal Screening and Recording (PSR) is a dental screening tool that is used to assess the health of the gums and teeth, and to identify any signs of gum disease, such as gingivitis and periodontitis. The PSR was performed by a dentist from our team. The PSR assessment involved a visual examination of the gums and teeth, as well as probing of the gums around each tooth to check for signs of gum disease. The probing is done using a special periodontal probe with a ball-shaped tip. The PSR is a screening tool that uses a scoring system to assess the health of the gums and teeth. The scoring system ranges from 0 to 4, with 0 indicating healthy gums and 4 indicating severe gum disease [Score 0: Healthy gums. No bleeding and pocket depth is within normal range (less than or equal to 3 mm); Score 1: Gingivitis. Bleeding on probing is present, but the pocket depth is within normal range (less than or equal to 3 mm); Score 2: Early periodontitis. Bleeding on prob-ing is present, and pocket depth is greater than 3 mm but less than or equal to 5 mm; Score 3: Moderate periodontitis. Bleeding on probing is present, and pocket depth is greater than 5 mm but less than or equal to 7 mm and Score 4: Severe periodontitis. Bleeding on prob-ing is present, and pocket depth is greater than 7 mm]. The PSR score for each tooth is based on the highest score recorded in any of the six sites around the tooth. The six sites include three on the buccal side of the tooth and three on the lingual side of the tooth. We used the worst sextant (highest score) to determine patient’s periodontal status [27].

### Statistical analysis

Statistical analyses were performed using SPSS 28.0 software considering an alpha level of 5% by an Applied Statistics Spec (PMC). Exploratory analysis included mean, standard deviation, median, percentages, and plots. No data treatment/imputation was performed. Chi-square and Kruskal-Wallis tests were applied to dichotomous and continuous variables, respectively.

The 60 food-items extracted from FFQ were reduced by means of principal component analysis (PCA). PCA with Oblimin rotation was used to estimate the components emerging and to obtain optimal correlated components. Initially, the correlation matrix of the standardized variables was examined, and the number of components to retain was based on eigenvalues, total of explained variance, and scree plot examination. The overall Kaiser–Meyer–Olkin (KMO) measure and Bartlett’s test of sphericity were examined as assumptions of the test.

Further, Fisher’s discriminant analysis was used to ascertain which of the aspects would be significant to discriminate the participants according to periodontal status. The following assumptions of the test were observed: independence of observations, multivariate normality, and homogeneity of variance (Box’s M statistic with a threshold of α = 1%). The following variables were considered in the analysis: sex, waist circumference, smoking habit, diabetes mellitus, recent periodontal treatment, and the regression coefficients of the components extracted from FFQ as described above. The stepwise method and Wilks’ Lambda were applied. Given the heterogeneity of the participants’ age, the comparison was performed within each age-range: 18–39 years (n = 97), 40–59 years (n = 234), and >60 years (elderly, n = 111).

## Results

The description of the studied population is shown in Table 1, according to age group and periodontal status subgroups. It is of note the prevalence of excess weight, which was higher than 65% in all age groups. BMI increased with increasing age, concomitant with an increase in the prevalence of chronic diseases. The periodontal status subgroups were homogeneous for sex and BMI, although a difference in schooling and waist circumference (18–39 years), and the presence of chronic diseases (40–59 years) were found (p<0.05).

**Table 1:** Sample characteristics, nutritional and food intake according to age range.

Age range	18–39 years			40–59 years			>60 years		
*Periodontal status*	*0*	*1*	*2*	*0*	*1*	*2*	*0*	*1*	*2*
*n*	*60*	*31*	*6*	*61*	*110*	*63*	*20*	*33*	*58*
**Clinical parameters**									
Sex (% females)	78	81	67	87	82	75	80	88	66
Schooling (% >8 years)	93*	74*	83*	51	55	40	30	46	35
BMI (kg/m^2^)	26.0 (4.3)	29.4 (7.3)	27.6 (5.9)	28.9 (6.5)	29.6 (6.4)	30.7 (6.6)	30.6 (4.1)	30.6 (5.2)	28.0 (5.6)
Normal/overweight/obesity/morbid obesity (%)	37/45/18/0	29/23/42/6.5	34/33/33/0	28/38/27/8	23/36/32/8	15/39/38/8	0/50/45/5	13/38/44/6	30/34/32/4
Waist circumference (cm)	82.6^A^ (10.1)	90.9^B^ (14.7)	88.4^AB^ (13.8)	89.9 (12.6)	91.6 (13.2)	95.8 (13.8)	94.8 (11.5)	93.5 (11.4)	94.3 (13.1)
Diabetes melittus (%)	5	16	0	21	26	33	40	58	47
Tobacco/Alcohol habit (%)	28	32	17	20	28	37	35	49	31
Other chronic diseases† (%)	23	32	50	53*	72*	64*	90	91	85
**Nutritional parameters**									
Energy (kcal/day)	1812.6 (482.4)	1655.6 (436.5)	1479.5 (315.2)	1556.6 (441.1)	1537.9 (414.0)	1577.0 (519.6)	1387.1 (367.0)	1332.5 (387.3)	1456.7 (406.1)
Protein (g/day)	78.5 (31.6)	82.6 (48.7)	61.7 (17.7)	64.2 (24.7)	70.5 (38.6)	69.0 (31.5)	60.9 (25.5)	59.2 (21.5)	64.7 (29.1)
Total carbohydrate (g/day)	266.4 (89.0)	249.5 (83.3)	227.5 (47.9)	226.0 (69.2)	234.0 (103.9)	232.7 (99.2)	208.1 (70.0)	194.2 (65.2)	216.8 (62.6)
Fats	58.1 (21.4)	56.8 (28.8)	46.5 (13.4)	49.4 (22.8)	49.9 (32.1)	47.1 (23.6)	42.3 (26.6)	42.0 (18.9)	45.8 (23.5)
SFA (g/day)	20.5 (8.2)	19.2 (9.7)	15.9 (5.4)	17.0 (8.5)	17.7 (11.2)	16.5 (8.8)	14.1 (8.1)	15.4 (7.9)	16.5 (9.0)
MUFA (g/day)	19.6 (7.1)	20.1 (10.9)	16.3 (5.3)	17.3 (8.2)	17.7 (11.5)	16.4 (8.7)	15.3 (10.5)	14.5 (6.8)	16.0 (8.8)
PUFA (g/day)	11.2 (5.2)	11.3 (6.1)	8.6 (2.2)	9.4 (4.8)	8.8 (6.5)	8.6 (4.2)	7.9 (6.0)	6.8 (3.2)	7.8 (4.1)

SFA—Saturated Fatty Acids; MUFA—Monounsaturated Fatty Acids; PUFA—Polyunsaturated Fatty Acids.

†Considered as the presence of (any) hypertension, hypercholesterolemia, hypertriglyceridemia, hypo/hyperthyroidism, steatosis, cardiopathy, renal or circulatory disease, depression.

*p<0.05 (Chi-square test). A≠B (p<0.05; Kruskal-Wallis test). Data are shown as mean and standard deviation for continuous variables and percentages for categorical variables (n = 442).

**Fig 1:**
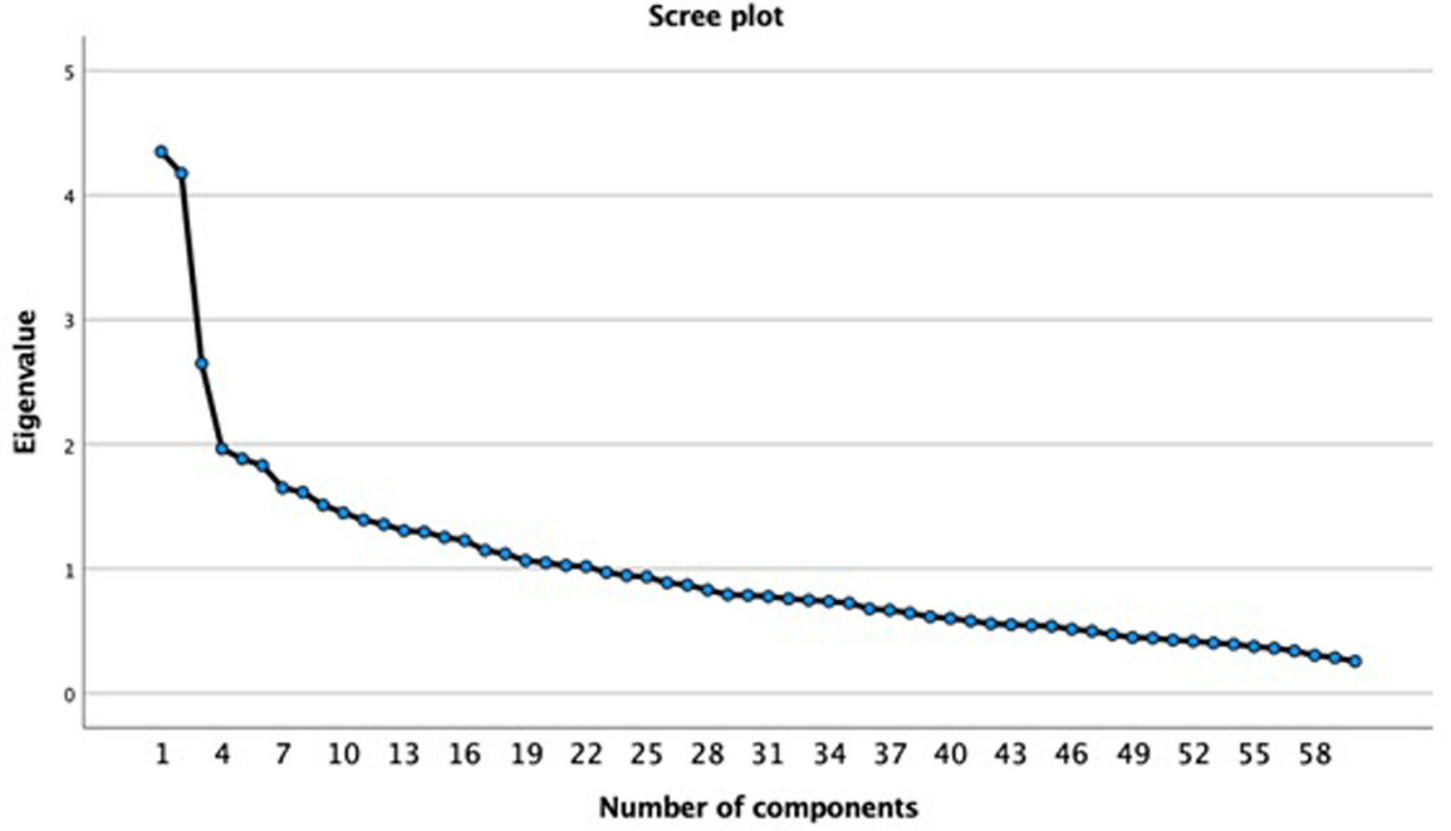
Scree plot used to examine the inflection point.

To estimate the food preferences from the intake of 60 food-items, PCA was performed as previously described resulting in 22 components (Fig 1 and S1 Table). The KMO measure was 0.68, and the Bartlett’ sphericity test was significant (P<0.0001). The 22 components explained 62.2% of total variance (S1 Fig), corresponding to the following dietary patterns: C1 with positive loadings for crackers, fries and processed meat; C2: green leaves, vegetables and olive oil; C3: negative loadings for chicken, beans and rice; C4: banana, pork, pork chop, tea and coffee; C5: negative loadings for pasta, yellow cheese, fresh cheese, ricotta and cake; C6: bread, toast, margarine; C7: brown rice, whole grain bread/toast; C8: negative loadings for roasted potato, manioc and yam, melon, watermelon, egg; C9: milk, cheese, chocolate powder; C10: yogurt (positive loading) and sandwich (negative loading); C11: fried fish, beans with pork (feijoada); C12: soup, pasta; C13: whole wheat flour or oats, papaya, orange, pear, canned peas; C14: guava, avocado, cassava flour; C15: negative loadings for vegetables with mayonnaise, chocolate, sweets; C16: bitter raw greens, broccoli, kale and cabbage; C17: fried or baked snacks, pizza, pancakes, soda; C18: corn meal; C19: olive oil, condiments; C20: pork, steak, beer, sausages; C21: negative loadings for orange, pineapple, banana, apple and pear and positive loading for industrialized juice; C22: negative loadings for red meat, sausage, pork, bacon, yellow cheese.

Finally, Fisher’s discriminant analysis was performed to ascertain which of the aspects would discriminate the participants according to periodontal status in each age group.

In the age group 18–39 years, one significant discriminant function was obtained (F) as the variable ‘waist circumference’ was the only significant predictor as follows: F = -7.108 +0.083*waist circumference (p = 0.010; Box’s M statistic = 5.905; p = 0.058). The healthier periodontal status (score 0) resulted from a lower waist circumference.

In the age group 40–59 years, two discriminant functions were obtained (F1 and F2), and 74% of the variance was explained by F1 (p = <0.001) (Box’s M statistic = 38.145; p = 0.011). According to F1, the standardized coefficients indicated that the variable ‘Component 2’ was the most important predictor, followed by Component 21, Component 1, and diabetes. The two functions are described below:

F1 = -0.460 +1.220*Diabetes -0.493*Component 1–0.726*Component 2 +0.657*Component 21

F2 = -0.019 +0.511*Diabetes +0.919*Component 1–0.077*Component 2 +0.504*Component 21

**Fig 2:**
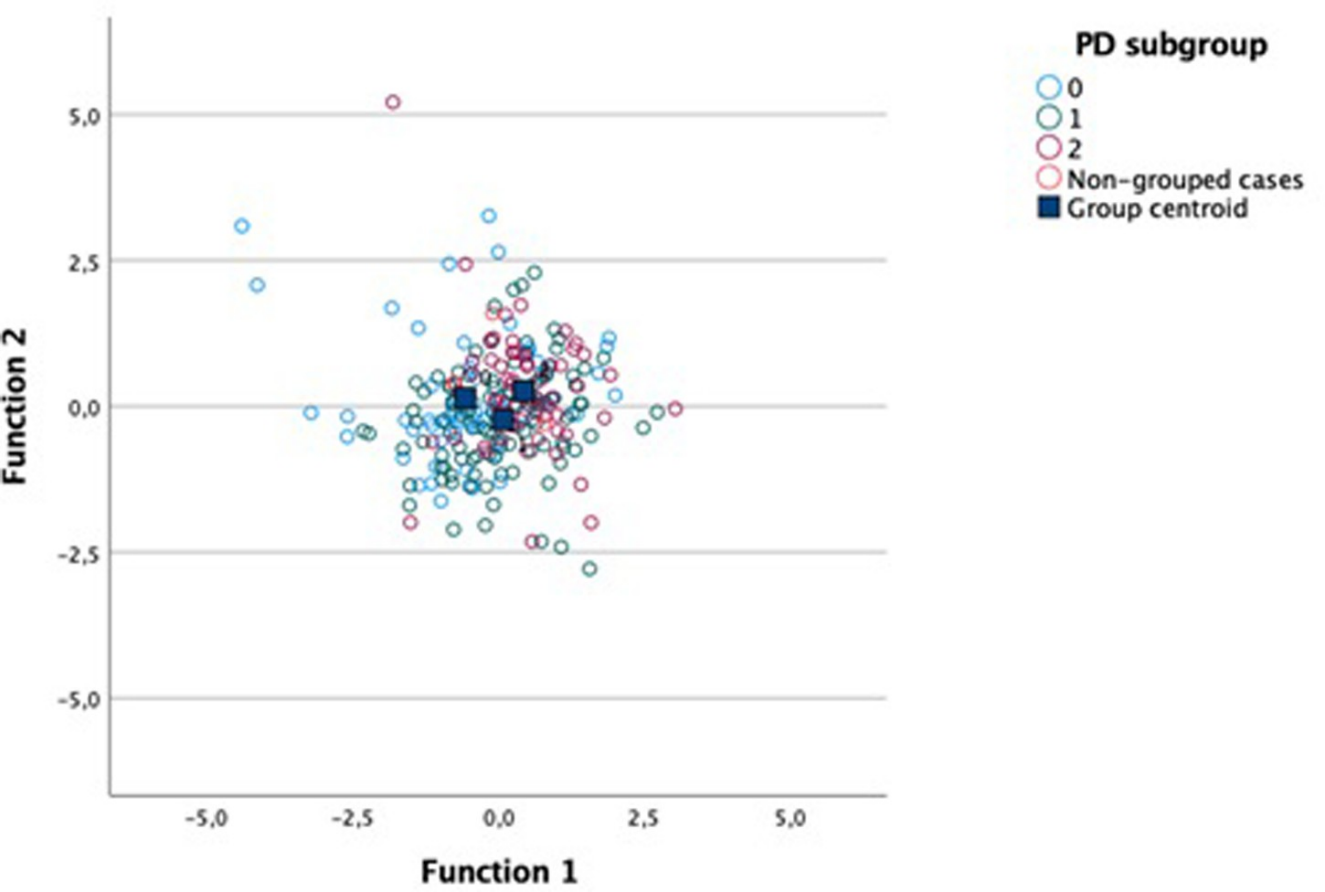
Graphical analysis illustrating the discriminatory power of the functions and centroids of the three periodontal disease (PD) subgroups in the age group of 40–59 years.

As observed in Fig 2, F1 discriminates better between the those with better periodontal status (score 0—blue dots) and the other two groups, while F2 helps to discriminate between intermediate periodontal status (score 1—green dots), and the other two groups. Based on the findings, the worst periodontal status (score 2) results from a higher frequency of diabetes, lower intake of green leaves, vegetables, and olive oil (Component 2), and fruit (Component 21), and higher intake of industrialized juice (Component 21). However, the healthier periodontal status results from a lower frequency of diabetes and higher intake of fruit and vegetables. Periodontal scores 1 and 2 also showed lower classification coefficients for the intake of crackers, fries and processed meat (Component 1).

In the age group >60 years, one significant discriminant function was obtained (F) as the variable ‘sex’ was the only significant predictor as follows: F = -1.774 +2.365*sex (p = 0.019; Box’s M statistic = 8.486; p = 0.015). The worst periodontal status (Score 2) resulted from a higher frequency of male sex.

## Discussion

This cross-sectional population-based study demonstrates that in general periodontal disease was closely associated with lower intake of green leaves, vegetables and olive oil, and fruit, and higher intake of industrialized juice. Additionally, male sex, increased waist circumference and the presence of chronic diseases such of diabetes in certain age groups were directly associated with worst periodontal status.

We found a high prevalence (65%) of overweight/obesity in the studied population, corroboration the National Primary Health Care and Anthropometric Survey conducted by the Ministry of Economy in 2019 [34]. This research showed that 60.3% of the Brazilian population is overweight, reaching 62.6% of women and 57.5% of adult men [34]. Periodontal disease, overweight/obesity and NCDs such as diabetes are closely related due to common risk factors, comprising especially excess sugar ingesting and communal inflammatory pathways [35]. In the present study BMI tended to increase with age contributing to the increase in waist circumference [36] which are known to be associated to periodontal disease [37,38]. In the age group of 18–39 years, the only significant predictor for periodontal status was ‘waist circumference’.

The low level of education was also associated with periodontal disease in the 18–39 age group, corroborating the idea that low educational achievement may increase the risk of periodontitis [39]. Despite this result, one may consider a further risk factor associated to poor social circumstances [40], since the present sample comprised people assisted by the public health system and socio-economic status is frequently associated with income and educational achievement [39].

In the age group 40–59 years, the worst periodontal status was associated to a higher frequency of diabetes, lower intake of green leaves, vegetables and olive oil, and fruit, and higher intake of industrialized juice. The bidirectional relationship between diabetes and periodontal diseases is well known in the literature [41]. Elevated blood glucose levels induce vascular alterations, impaired immune response and delays in tissue repair. On the other hand, the chronic release of pro-inflammatory cytokines in active periodontal disease reduces insulin action [42,43].

In relation to diet, the consumption of at least 5 servings of fruits and vegetables was found as capable of preventing the progression of periodontal diseases, especially periodontitis, and even tooth loss due to the greater presence of vitamins, minerals and polyphenolic compounds [44]. Industrialized juice is rich in sugar, and high-glycemic, processed products (like sugary drinks) can be major causes of chronic inflammation [45–47].

Surprisingly, worst periodontal status was also associated to a low consumption of crackers, fries, and processed meat, probably because of the higher frequency of diabetes and other NCDs in these subgroups, respectively. Processed meats, ready-to-eat meals, and packaged snacks are known to be associated with NCDs [48,49]. However, meat tends to be expensive and consumption in the present sample was low. Food prices are one of the most important determinants of food choices, in populations with low-socioeconomic status [50,51]. Besides, the Family Health Strategy employ group dynamics, physical activity practices and nutritional education follow-ups especially devoted for diabetic and hypertense patients [52], which may have contributed to better food choices in this group.

Considering the age group >60 years-old the only significant predictor for worse periodontal condition was male sex. A previous epidemiological study conducted in Brazil found that periodontal disease was more prevalent in older adults, brown-skinned, male, with lower family income and lower schooling [53] which is in accordance to the present findings. Age and male sex have already been reported to be positively associated with the prevalence of several periodontal pathogens both in periodontal pockets and in saliva [54].

The existence of methodological disparities in diagnosing periodontal disease poses a significant challenge when comparing findings across different studies. Depending on how periodontitis is defined or measured, various results can be obtained when examining its associations with other systemic health conditions. Regarding the diagnosis of periodontal disease in the present study, we opted not to utilize the new classification from 2018 [55], which involves a multidimensional evaluation besides staging and grading periodontal disease. Despite the widespread adoption of the new classification in the literature since its release in 2018, there are still difficulties in its application in population-based studies [56]. Besides, the assessment of dietary intake presents its own set of encounters as well and can be accomplished using various methodologies. Food frequency questionnaires (FFQ) are valuable for assessing habitual dietary intake and prove to be cost-effective and time-efficient, making them suitable for use in epidemiological studies [57].

The results of this study have important implications for the Brazilian healthcare system, particularly for the provision of preventative dental care. The findings suggest that promoting healthy dietary habits could help prevent periodontal disease, which is a major cause of tooth loss and can have serious consequences for overall health. Therefore, public health policies and programs should prioritize the promotion of healthy diets and provide access to nutritional education and counseling to individuals who receive care through the public health system. Furthermore, dental professionals should incorporate nutrition-al assessments and counseling into routine dental care, particularly for patients at risk for periodontal disease, to improve patient outcomes and prevent costly interventions. Future research should focus on the development of interventions aimed at promoting healthy dietary habits and improving periodontal health status among adults who receive care through the public health system. One potential avenue for research is the design and implementation of nutrition education programs that target specific populations based on age, sex, and chronic disease status. These programs could focus on promoting the con-sumption of natural and minimally processed foods, increasing the intake of fruits and vegetables, and reducing the consumption of processed and ultra-processed foods. Additionally, future research could explore the potential role of dietary supplements, such as vitamins, prebiotics/probiotics and minerals, in combination with dietary interventions to improve periodontal health status in accordance with international guidelines/recommendations [58].

In conclusion, a low preference of natural foods associated to a high intake of processed and ultra-processed foods, together with high waist circumference, older age, male sex, and diabetes might influence periodontal health status in adults assisted by a public health care system.

## Supporting information

S1 Fig Component loadings of food intake patterns obtained by principal component analysis with Oblimin rotation. Only coefficients higher than 0.30 are shown. Rotation converged with 58 interactions.
(DOCX)

S1 Table (XLSX)
