# Supplementary material for: Food preferences and periodontal status of adults assisted by a public health care system
Source: PLoS One. 2023 Oct 18;18(10):e0291878. doi: 10.1371/journal.pone.0291878 (PMC10584113; doi:10.1371/journal.pone.0291878)
Supplement: S1 Fig — Only coefficients higher than 0.30 are shown. Rotation converged with 58 interactions. (DOCX) [file pone.0291878.s001.docx]

**S1 Fig.** Component loadings of food intake patterns obtained by principal component analysis with Oblimin rotation. Only coefficients higher than 0.30 are shown. Rotation converged with 58 interactions.

| **Component** | **1** | **2** | **3** | **4** | **5** | **6** | **7** | **8** | **9** | **10** | **11** | **12** | **13** | **14** | **15** | **16** | **17** | **18** | **19** | **20** | **21** | **22** |
| --- | --- | --- | --- | --- | --- | --- | --- | --- | --- | --- | --- | --- | --- | --- | --- | --- | --- | --- | --- | --- | --- | --- |
| *Explained variance (%)* | *7.3* | *14.2* | *17* | *22* | *25* | *28* | *31* | *33.5* | *36* | *38.5* | *41* | *43* | *45.2* | *47.4* | *49.5* | *51.5* | *53.4* | *55.3* | *57.1* | *59* | *60.5* | *62.2* |
| Boiled or fried corn meal |  |  |  |  |  |  |  |  |  |  |  |  |  |  |  |  |  | -.731 |  |  |  |  |
| Milk |  |  |  |  |  |  |  |  | .562 |  |  |  |  |  |  |  |  |  |  |  |  |  |
| Oat flour or bran or whole oats |  |  |  |  |  |  |  |  |  |  |  |  | .806 |  |  |  |  |  |  |  |  |  |
| Lettuce |  | .731 |  |  |  |  |  |  |  |  |  |  |  |  |  |  |  |  |  |  |  |  |
| Tomato |  | .829 |  |  |  |  |  |  |  |  |  |  |  |  |  |  |  |  |  |  |  |  |
| Carrot |  | .506 |  |  |  |  |  |  |  |  |  |  |  |  |  |  |  |  |  |  |  |  |
| Other vegetables (zucchini, chayote, cucumber) |  | .347 |  |  |  |  |  |  |  |  |  |  |  |  |  |  |  |  |  |  |  |  |
| Other raw vegetables (chard, arugula, watercress) |  |  |  |  |  |  |  |  |  |  |  |  |  |  |  | .585 |  |  |  |  |  |  |
| Potato, cassava, yam (boiled or roasted) |  |  |  |  |  |  |  | -.405 |  |  |  | .337 |  |  |  |  |  |  |  |  |  |  |
| Broccoli, Cauliflower, Cabbage |  |  |  |  |  |  |  |  |  |  |  |  |  |  |  | .784 |  |  |  |  |  |  |
| Orange, tangerine, pineapple |  |  |  |  |  |  |  |  |  |  |  |  |  |  |  |  |  |  |  |  | -.678 |  |
| Banana |  |  |  | .369 |  |  |  |  |  |  |  |  |  |  |  |  |  |  |  |  | -.383 |  |
| Apple, pear |  |  |  |  |  |  |  |  |  |  |  |  |  |  |  |  |  |  |  |  | -.499 |  |
| Melon, watermelon |  |  |  |  |  |  |  | -.658 |  |  |  |  |  |  |  |  |  |  |  |  |  |  |
| Papaya |  |  |  |  |  |  |  |  |  |  |  |  | .513 |  |  |  |  |  |  |  |  |  |
| Guava |  |  |  |  |  |  |  |  |  |  |  |  |  | .623 |  |  |  |  |  |  |  |  |
| Avocado |  |  |  |  |  |  |  |  |  |  |  |  |  | .604 |  |  |  |  |  |  |  |  |
| chicken breast soup with noodles, vegetables |  |  |  |  |  |  |  |  |  |  |  | .770 |  |  |  |  |  |  |  |  |  |  |
| Pasta with meatless sauce |  |  |  |  |  |  |  |  |  |  |  | .505 |  |  |  |  |  |  |  |  |  |  |
| Pasta, bolognese sauce |  |  |  |  | -.748 |  |  |  |  |  |  |  |  |  |  |  |  |  |  |  |  |  |
| Beef, striploin with fat, grilled |  |  |  |  |  |  |  |  |  |  |  |  |  |  |  |  |  |  |  |  |  | -.396 |
| Pork, grilled steak |  |  |  | .328 |  |  |  |  |  |  |  |  |  |  |  |  |  |  |  | .358 |  |  |
| Sausage, pork, grilled |  |  |  |  |  |  |  |  |  |  |  |  |  |  |  |  |  |  |  |  |  | -.671 |
| Chicken, thigh with skin, braised |  |  | -.613 |  |  |  |  | -.308 |  |  |  |  |  |  |  |  |  |  |  |  |  |  |
| Fry fish fillet |  |  |  |  |  |  |  |  |  |  | .769 |  |  |  |  |  |  |  |  |  |  |  |
| Chicken egg, fried |  |  |  |  |  |  |  | -.720 |  |  |  |  |  |  |  |  |  |  |  |  |  |  |
| Baked beans |  |  | -.758 |  |  |  |  |  |  |  |  |  |  |  |  |  |  |  |  |  |  |  |
| Beans with pork (feijoada) |  |  |  |  |  |  |  |  |  |  | .625 |  |  |  |  |  |  |  |  |  |  |  |
| Cooked rice |  |  | -.669 |  |  |  |  |  |  |  |  |  |  |  |  |  |  |  |  |  |  |  |
| Cooked brown rice |  |  |  |  |  |  | .837 |  |  |  |  |  |  |  |  |  |  |  |  |  |  |  |
| French fries | .400 |  |  |  |  |  |  |  |  |  |  |  |  |  |  |  |  |  |  |  |  |  |
| Vegetable salad with mayonnaise |  |  |  |  |  |  |  |  |  |  |  |  |  |  | -.355 |  |  |  |  |  |  | -.311 |
| Cabbage braised in butter |  |  |  |  |  |  |  |  |  |  |  |  |  |  |  | .650 |  |  |  |  |  |  |
| Orange, pear, juice |  |  |  |  |  |  |  |  |  |  |  |  | .319 |  |  |  |  | .323 |  |  |  |  |
| Olive oil |  | .509 |  |  |  |  |  |  |  |  |  |  |  |  |  |  |  |  | .421 |  |  |  |
| Bacon |  |  |  |  |  |  |  |  |  |  |  |  |  |  |  |  |  |  |  |  |  | -.751 |
| Mozzarella cheese, plate, parmesan, provolone |  |  |  |  | -.324 |  |  |  |  |  |  |  |  |  |  |  |  |  |  |  |  | -.347 |
| Fresh cheese, ricotta |  |  |  |  | -.412 |  |  |  | .334 | .323 |  |  |  |  |  |  |  |  |  |  |  |  |
| Canned, canned peas |  |  |  |  |  |  |  |  |  |  |  |  | .799 |  |  |  |  |  |  |  |  |  |
| Beer | -.345 |  |  |  |  |  |  |  |  |  |  |  |  |  |  |  |  |  |  | .397 |  |  |
| French bread, toast |  |  |  |  |  | .687 |  |  |  |  |  |  |  |  |  |  |  |  |  |  |  |  |
| Fried snacks |  |  |  |  |  |  |  |  |  |  |  |  |  |  |  |  | .658 |  |  |  |  |  |
| Baked snacks |  |  |  |  |  |  |  |  |  |  |  |  |  |  |  |  | .791 |  |  |  |  |  |
| Pizza, pancake |  |  |  |  |  |  |  |  |  |  |  |  |  |  |  |  | .518 |  |  |  |  |  |
| Sausages (ham, mortadella, sausage) |  |  |  |  |  |  |  |  |  |  |  |  |  |  |  |  |  |  |  | .761 |  |  |
| Hamburger, nuggets, meatball | .405 |  |  |  |  |  |  |  |  |  |  |  |  |  |  |  |  |  |  |  |  |  |
| Yogurt flavored with fruit |  |  |  |  |  |  |  |  |  | .445 |  |  |  |  |  |  |  |  |  |  |  |  |
| Ready-made cassava flour |  |  |  |  |  |  |  |  |  |  |  |  |  | .545 |  |  |  |  |  |  |  |  |
| Spice |  |  |  |  |  |  |  |  |  |  |  |  |  |  |  |  |  |  | .824 |  |  |  |
| Industrialized juice |  |  |  |  |  |  |  |  |  |  |  |  |  |  |  |  |  |  |  |  | .613 |  |
| Refrigerator |  |  |  |  |  |  |  |  |  |  |  |  |  |  |  |  | .362 |  |  |  |  |  |
| Bread, wholemeal, wholemeal toast |  |  |  |  |  |  | .550 |  |  |  |  |  |  |  |  |  |  |  |  |  |  |  |
| Wafer biscuit | .715 |  |  |  |  |  |  |  |  |  |  |  |  |  |  |  |  |  |  |  |  |  |
| Ready-made cake |  |  |  |  | -.752 |  |  |  |  |  |  |  |  |  |  |  |  |  |  |  |  |  |
| Margarine spread on bread |  |  |  |  |  | .779 |  |  |  |  |  |  |  |  |  |  |  |  |  |  |  |  |
| Sandwich and hot dog |  |  |  |  |  |  |  |  |  | -.481 |  |  |  |  |  |  |  |  |  |  |  |  |
| Chocolate, bonbon |  |  |  |  |  |  |  |  |  |  |  |  |  |  | -.646 |  |  |  |  |  |  |  |
| Chocolate powder (added to milk) |  |  |  |  |  |  |  |  | .772 |  |  |  |  |  |  |  |  |  |  |  |  |  |
| Desserts, milk jam |  |  |  |  |  |  |  |  |  |  |  |  |  |  | -.740 |  |  |  |  |  |  |  |
| tea and coffee |  |  |  | .659 |  |  |  |  |  |  |  |  |  |  |  |  |  |  |  |  |  |  |
